# Sterilization by Ozone: Effects on Electrospun Polycaprolactone Membrane Properties and Cell Viability

**DOI:** 10.1155/ijbm/9230130

**Published:** 2025-12-28

**Authors:** Taisa L. S. Farias, Ivanildes Bastos, Joelma Cavalcante Ricardo, Jessica F. Cunha, Yonny Romaguera-Barcelay, Ariamna Gandarilla, Karen Segala, Patrícia Puccinelli Orlandi, Rúben Fernandes, Marcos Marques da Silva Paula, Walter Ricardo Brito

**Affiliations:** ^1^ Department of Chemistry, Federal University of Amazonas, Manaus, Amazonas, Brazil, ufam.edu.br; ^2^ Leônidas and Maria Deane Institute, Fiocruz Foundation, Manaus, Amazonas, Brazil; ^3^ Department of Functional Biology and Health Sciences, Faculty of Biology, University of Vigo, Pontevedra, Spain, uvigo.gal; ^4^ Center of Clinical Studies, Fernando Pessoa Hospital, Gondomar, Portugal; ^5^ Instituto Aggeu Magalhães, Fiocruz Foundation, Recife, Pernambuco, Brazil; ^6^ Department of Physical Engineering, Federal University of Amazonas, Manaus, Amazonas, Brazil, ufam.edu.br

## Abstract

This study focuses on developing and characterizing electrospun polycaprolactone (PCL) membranes as scaffolds for cell growth, leveraging their ability to mimic the extracellular matrix and promote cell proliferation. The membranes were fabricated by electrospinning and sterilized using ozone at room temperature. Comprehensive characterization techniques, including scanning electron microscopy (SEM), contact angle measurements, ultraviolet–visible spectroscopy (UV–Vis), differential scanning calorimetry (DSC), thermogravimetric analysis (TGA), Fourier transform infrared (FTIR) spectroscopy, Raman spectroscopy, and in vitro biocompatibility assays with MRC‐5 cells, were employed. The electrospun membranes exhibited uniform fibers with an average diameter of 403 ± 100 nm and demonstrated sterility, with no microbial growth observed after incubation. Contact angle measurements revealed values of 123 ± 0.42° and 123 ± 0.25° for nonsterilized and sterilized membranes, respectively, indicating consistent hydrophobicity. Thermal analyses confirmed the structural stability of PCL membranes, while UV–Vis studies validated their controlled degradation and release kinetics. FTIR and Raman spectroscopy confirmed that ozone sterilization preserved the chemical integrity of the membranes, with no new organic functions observed. Biocompatibility assays demonstrated high cell viability (> 97%) and effective adhesion on the membranes, highlighting their compatibility and suitability for supporting cell proliferation. These results demonstrate the efficacy of ozone sterilization and the potential of electrospun PCL membranes for a wide range of biomedical applications, including tissue engineering, wound healing, and drug delivery systems.

## 1. Introduction

Resorbable polymers have been extensively studied for their ability to combine adjustable properties with biodegradability, characteristics that are particularly important in biomaterials. Among these polymers, poly(ε‐caprolactone) (PCL) has emerged as one of the most promising materials due to its controlled degradation, which occurs throughout 2–4 years [1]. This feature makes it ideal for tissue engineering applications, where a temporary scaffold can support cell growth and be gradually resorbed by the body [2]. PCL is a synthetic aliphatic polyester with remarkable thermal and mechanical properties, such as a relatively low melting point (59°C–64°C) and a glass transition temperature of approximately −60°C [3]. In addition, PCL is known for its high biocompatibility, excellent blend ability with other polymers, and significant thermal stability, which contribute to its versatility in biomedical applications [1–3].

Electrospinning has been widely explored among the various processing techniques for PCL due to its ability to produce continuous nanofiber membranes with controlled morphology. This technique enables the development of three‐dimensional porous scaffolds that are highly effective in supporting cell growth and promoting enhanced interactions between cells and their extracellular environment. The increased surface area provided by nanofibers facilitates protein adsorption and interactions with cell receptors, which are crucial for tissue regeneration and controlled drug delivery applications [4]. Furthermore, electrospinning has demonstrated excellent results in the production of advanced wound dressings, where the membranes can accelerate the healing process due to their controlled microarchitecture and specific properties [5].

Ensuring sterility is one of the most critical challenges in electrospun membranes, as this requirement is essential to prevent contamination during clinical use. In this context, ozone has emerged as a promising sterilization method. Its oxidative properties enable the efficient elimination of a wide range of microorganisms without significantly degrading the material’s physical and chemical characteristics. Moreover, ozone does not leave toxic residues, making it highly attractive for sensitive medical device applications [6]. Compared to traditional sterilization methods, such as gamma radiation or chemical treatments, which often compromise the structural integrity of fibers or leave undesirable residues [6], ozone offers an alternative that maintains the functional and morphological integrity of electrospun scaffolds [7].

This study investigated ozone as a sterilizing agent for electrospun PCL membranes. Previous studies suggest that ozone application minimizes the degradation of electrospun fibers, thereby preserving their morphological and functional integrity [8]. However, optimizing process parameters was required to ensure the sterilization process was effective and to meet the specific requirements of medical devices. In this work, we adapted the sterilization method to the available equipment by adjusting gas concentration, exposure time, and the number of ozone pulses applied. These adjustments were made to maximize the method’s effectiveness without compromising the membranes′ physical, chemical, or biological properties.

In addition to sterilization, a fundamental aspect of this study was the analysis of the membranes′ biocompatibility with MRC‐5 cells, a human lung fibroblast [9]. This cell model is widely used in studies of cell adhesion and viability in biomaterials. The interactions between cells and membranes were evaluated to verify the preservation of the material’s functionality after sterilization. The developed membranes were characterized by their morphology using scanning electron microscopy (SEM), chemical properties using Fourier transform infrared (FTIR) spectroscopy and Raman spectroscopy, and wettability via contact angle measurements.

Finally, the results of this study highlight the potential of ozone‐sterilized electrospun PCL membranes not only for their efficacy in eliminating microorganisms but also for maintaining properties that make them suitable for supporting cell growth. These advancements offer new perspectives for applications in sensitive medical areas, such as wound dressings and tissue regeneration scaffolds.

## 2. Materials and Methods

### 2.1. Materials

All chemicals used in this study are analytically pure and used directly without further purification. PCL with a molecular weight of 80,000 g/mol was purchased from Sigma‐Aldrich. Solvents such as chloroform (99.0% purity) and acetone (99.8% purity) were acquired from Biotec. The syringes used were model SR 10 mL with 0.8‐mm needles.

### 2.2. Electrospinning Setup

Firstly, a 1:1 mass ratio of solvents (acetone and chloroform) was prepared, followed by mechanical stirring for 15 min. Subsequently, polycaprolactone (PCL) was added to the solvent mixture at 13.6% (w/w), a concentration commonly used in electrospinning studies to achieve suitable viscosity and fiber formation. The solution was mechanically stirred for 4 h to ensure complete dissolution of the polymer. This concentration was chosen in accordance with previous work by Moraes Segundo et al. (2020), which successfully employed similar PCL solutions in electrospinning applications [10].

**Scheme 1:**
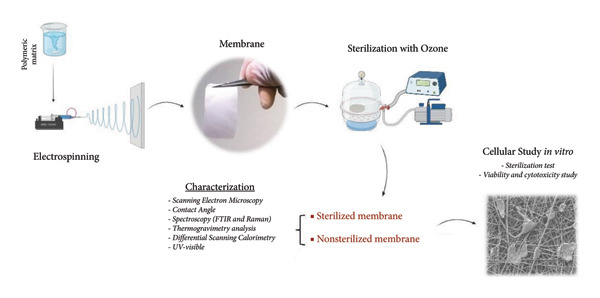
Representation of the manufacturing process and characterization of polymeric membranes. Original figure created by the authors.

The electrospinning system consists of a high‐voltage power supply (Faísca FA‐30 KV), an infusion pump (Bonther, Serie Touch), and a static aluminum collector (Scheme 1). The electrospinning parameters were defined based on the work of Farias et al. [1]. The environmental parameters were 22°C and 48% relative humidity. The process parameters were as follows: applied voltage of 13.3 kV, working distance of 16 cm, needle diameter of 0.8 mm, and flow rate of 8 mL/h.

### 2.3. Characterization Studies

SEM images were obtained using a Tescan Vega 3 microscope. The samples were gold‐coated using a Baltec sputter coater, model CPD 050. Based on the statistical study of fiber diameter conducted by Moraes Segundo [10], fiber diameters were measured using the DiameterJ plugin of ImageJ, recording 100 fiber diameters [11].

The wetting assay was performed by carefully depositing a 10‐μL drop of deionized water on the membrane surface at room temperature. The behavior of this drop was observed for 120 s using a digital microscope (DINO‐Lite Plus) with a magnification capacity of up to 1000×. Contact angle measurements were obtained using ImageJ.

A UV–Vis spectrophotometer (Evolution 220—Thermo Scientific, with a dual beam and fiber optic probe) was used to observe the PCL release profile. Quartz cuvettes with a 1‐cm optical path were used. To evaluate the release behavior of UV‐active compounds from PCL membranes into aqueous media, UV–Vis absorbance spectra were recorded at various immersion times (30–180 min) in Milli‐Q water. Separate experiments were conducted for sterilized and nonsterilized PCL membranes. The time‐dependent absorbance data were fitted to two kinetic models: a zero‐order model representing constant‐rate release (Equation (1)) and a first‐order model, representing concentration‐dependent release (Equation (2))

(1) At=k × t+Ainit,

(2) At=A0×1 − e−k.t.

Model fitting was performed using nonlinear regression, in which the rate constant *k* and other parameters were extracted by minimizing the sum of squared residuals. The goodness of fit was evaluated using the coefficient of determination (R^2^), residual sum of squares (RSS), and mean squared error (MSE). The methodology established practices in kinetic analysis of biomaterials as described by Higuchi (1963) for diffusion‐based release kinetics [12], Siepmann and Peppas (2001) for mechanistic modeling [13], and recent works focusing on PCL stability and degradation behavior in biomedical applications [14, 15].

Differential scanning calorimetry (DSC) analysis was performed using a NETZSCH DSC 204F1 Phoenix instrument. A sample with a mass of 5.5119 mg was placed in a concave aluminum crucible with a pierced lid. The purge gas flow consisted of an air mixture (80/20) at 250.3 mL/min and nitrogen at 250.0 mL/min, with an additional protective nitrogen flow at 250.0 mL/min. The analysis was conducted over a temperature range from −95°C to 190°C, with a heating rate of 5.0 K/min. Data were recorded in exothermic mode (EXO) with positive heat flow.

TGA was carried out using a NETZSCH TG 209F1 Libra instrument. A sample weighing 7.1463 mg was measured in an open Al_2_O_3_ crucible (85 μL). The primary purge gas flow consisted of an air mixture (80/20) at 250.3 mL/min and nitrogen at 250.0 mL/min, with nitrogen also serving as a protective gas at the same flow rate. The analysis spanned the temperature range from 30°C to 600°C with a controlled heating rate of 10.0 K/min.

FTIR spectra were recorded using an Agilent Cary 630 spectrometer, configured for 128 scans within a range of 4000 to 650 cm^−1^ at a resolution of 8 cm^−1^. Raman spectroscopy was conducted using a Renishaw inVia spectrometer operating in regular mode coupled to a microscope. A 514.5‐nm argon‐ion laser served as the excitation source, with the laser beam focused through a Leica objective featuring 50× magnification and a high numerical aperture (*N* = 0.5). Raman scattering measurements used a diffraction grating with 2400 lines per millimeter, and spectra were collected over the range 3200 to 100 cm^−1^.

The mechanical properties of electrospun PCL samples were evaluated using an INSPEKT Solo 2.5 mechanical testing system equipped with a 500 N load cell. Before testing, the samples were carefully cut into rectangular strips measuring 40 mm × 10 mm, and their thickness was precisely measured using a micrometer. Samples were securely positioned within the grips, held firmly by the specimen holders, and subjected to tensile loading at a crosshead speed of 5‐mm/min until mechanical failure. Each sample was tested in five replicates, and the results are presented as mean ± standard deviation (SD).

### 2.4. Ozone Sterilization

An Aldious ozone generator was utilized for sterilization, operating at a flow rate of 6.5 L/min and a maximum concentration of 150 ppm. Membranes were cut into 10 mm diameter discs and placed inside a desiccator equipped with a vacuum gauge. A vacuum was applied using a pump, and during the aeration phase, ozone gas was introduced at the generator’s maximum concentration, achieving a vacuum level of −50 mmHg, which was held for 5 min. This cycle was repeated four times, with 5‐min intervals between cycles. After sterilization, the membranes were removed from the desiccator and vacuum‐sealed using a Registron RG‐300A vacuum sealer.

The methodology employed was adapted from Rediguieri et al. [16], to optimize the sterilization process, considering the specific characteristics of the equipment used. Adjustments were made to parameters such as ozone gas concentration, flow rate, and the number of pulses applied, ensuring appropriate conditions for maximum efficiency and reproducibility.

#### 2.4.1. Biological Sterility Test

Sterility tests were conducted to quantify the presence and absence of microorganisms, including bacteria and fungi, in the membranes. The membranes were transferred to liquid bacterial growth media, Brain Heart Infusion (BHI) (HIMEDIA), Müeller Hinton (MH) (HIMEDIA), and to Sabouraud broth for fungi. The tubes containing 10 mL of the respective media were kept under agitation (150 rpm) for 72 h at 37°C for bacteria and 7 days at 27°C for fungi. Following the incubation period, media exhibiting turbidity indicated contamination by bacteria or fungi. Subsequently, 100 μL from each broth were plated onto different media: MacConkey agar (AMC) (HIMEDIA), Pseudomonas agar (PIA) (BD), Blood agar (AS) (HIMEDIA), and Mueller‐Hinton agar (MH) for bacterial growth, and Sabouraud agar for fungal growth. The plates were then incubated at 37°C for 72 h for bacteria and at 27°C for 7 days for fungi in a bacteriological oven.

#### 2.4.2. Human Lung Fibroblast (MRC‐5) Culture

For the evaluation of viability and cytotoxicity, a normal adherent human lung fibroblast cell line, MRC‐5, was used, obtained from the Rio de Janeiro Cell Bank (https://bcrj.org.br/celula/mrc-5?code=0180). This cell line is part of and stored at the Bioassay Platform for Biotechnological Compounds (RPT11H) of the Leônidas and Maria Deane Institute (ILMD).

The normal adherent human lung fibroblast cell line (MRC‐5) was cultured in Dulbecco’s modified Eagle’s medium (DMEM) culture medium (Sigma‐Aldrich, Brazil) supplemented with 10% fetal bovine serum (Sigma‐Aldrich, Brazil) and gentamicin (50 mg/L) (Sigma‐Aldrich, Brazil). Cells were maintained in an incubator at 37°C with 5% CO_2_. After reaching the desired confluence, the cells were plated at 1.0 × 10^4^ cells per well in 24‐well plates, with a final volume of 2 mL per well, and incubated for 24 h at 37°C.

The membranes were wrapped in glass coverslips for SEM assays and positioned at the bottom of the 24‐well plates. Then, 2 mL of DMEM containing a cell concentration of 1.0 × 10^4^ cells per well was added. The plates were then incubated for 48 h to allow cell adhesion and interaction with the membranes before analysis.

#### 2.4.3. Evaluation of Viability and Cytotoxicity of Human Lung Fibroblasts (MRC‐5)

The assays were conducted using the Alamar Blue method, as described by Souza et al. [17], with adaptations made by the Bioassay Platform Laboratory for Biotechnological Compounds (RPT11H) at the Leônidas and Maria Deane Institute (ILMD). All assays were performed in triplicate (*n* = 3 wells per condition) and repeated in three independent experiments to ensure reproducibility and statistical reliability.

After 24 h of incubation and adhesion, sterilized PCL membranes (PCLE) and nonsterilized PCL membranes (PCLNE) were added to each well and incubated at 37°C under 5% CO_2_ for 48 h. The viability and cytotoxicity of the membranes were evaluated by UV–Vis analysis of their profile release. After 48 h, the membranes were removed, and 100 μL of 0.1% Alamar Blue (Sigma‐Aldrich, Brazil) diluted 1:20 was added to each well. The plates were incubated for 2 h to allow Alamar Blue to metabolize, after which the reading was performed. Fluorescence was monitored using a microplate reader (GloMax® Explorer) with 580–640 nm emission wavelength and excitation at 520 nm. Cell viability results were expressed relative to the control group, consisting of MRC‐5 cells cultured in standard DMEM without membrane contact (cells alone + culture plate), representing 100% viability.

## 3. Results and Discussion

### 3.1. Morphological Study

Figure 1 displays SEM micrographs of fibers with a uniform, randomly distributed morphology and no pearl formation. As noted by Gonçalves [18], the production of defect‐free fibers relies on the proper regulation of surface tension and solvent evaporation rates, thereby facilitating optimal polymer‐solvent interactions. Additionally, no noticeable changes in the morphological properties of the fibers were observed after the electrospinning process. This outcome aligns with Rediguieri et al. [16], who studied the effects of ozone on PCL fibers and reported similar results.

Figure 1Fibers of PCL membranes: (a) nonsterilized and (b) sterilized. Statistics of PCL fiber diameters (c) nonsterilized and (d) sterilized.

**Figure:**
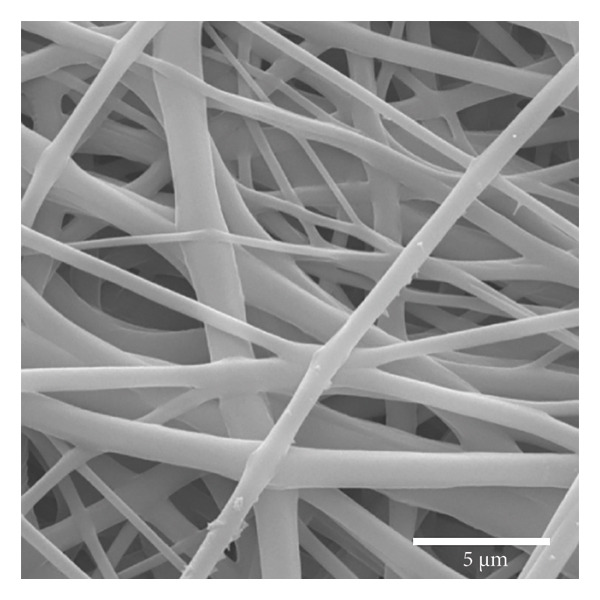
(a)

**Figure:**
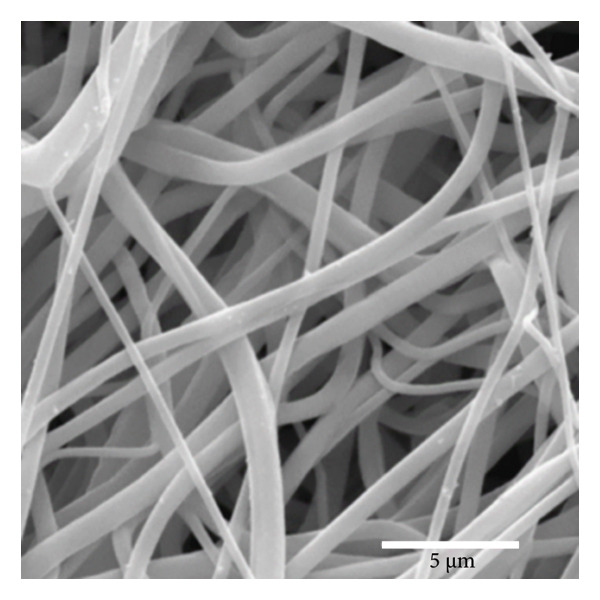
(b)

**Figure:**
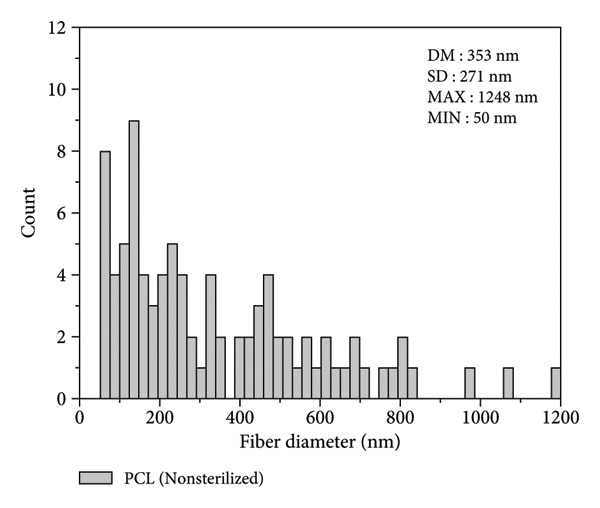
(c)

**Figure:**
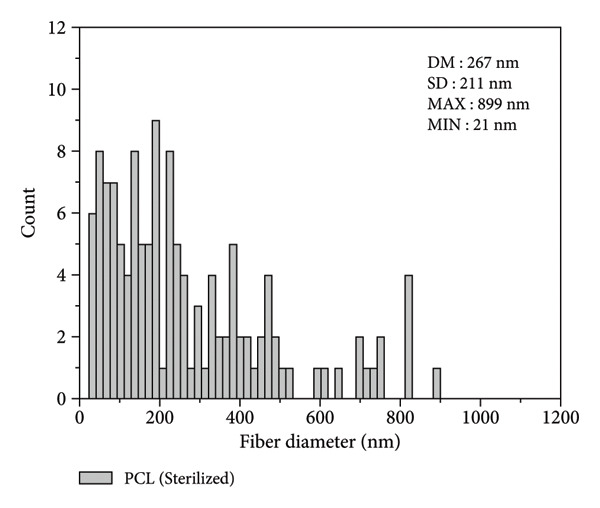
(d)

Figure 1(c) shows that the fiber diameters peak in the 80–620 nm range with an average diameter of DM = 353 nm and a standard deviation of SD = 271 nm. The maximum diameter found was 1248 nm, and the minimum was 50 nm. The statistical study of the diameter of sterilized fibers, presented in Figure 1(d), shows that the fiber diameters peak in the 60–470 nm range, with an average diameter of DM = 267 nm and a standard deviation of SD = 211 nm. The maximum diameter found was 899 nm, and the minimum was 21 nm. The results indicate that the nonsterilized fibers showed a 34% reduction in diameter after sterilization. However, the fibers demonstrated good distribution in all regions analyzed, showing an excellent correlation with the results obtained by Brito et al. [10]. Both sterilized and nonsterilized fibers showed uniform diameter distribution, confirming the efficiency of the electrospinning process. The slight decrease in average diameter after ozone sterilization should be interpreted with caution, as several factors can affect fiber formation. While Rediguieri et al. (2017) reported no morphological changes in PCL fibers exposed to ozone, Dabasinskaite et al. (2021) observed minor reductions attributed to surface oxidation and partial chain scission [16, 19]. The broad diameter range in both groups likely reflects the intrinsic variability of electrospinning, influenced by fluctuations in viscosity, solvent evaporation, and environmental conditions. Thus, the apparent decrease in diameter may result from both process variability and subtle oxidative effects of ozone.

### 3.2. Wettability Study

Table 1 presents the surface contact angles of the membranes to determine the wettability of PCL, which showed a contact angle of 123 ± 0.42° before sterilization and 122 ± 0.25° after sterilization. Low surface free energy levels characterize PCL. In other words, it is hydrophobic; therefore, the contact angle was expected to be greater than 90° [8, 10].

**Table 1:** Wettability of PCL membrane surfaces sterilized and nonsterilized.

Sample	Contact angle (°)
Nonsterilized	123 ± 0.42
Sterilized	122 ± 0.25

Studies report that the use of ozone for sterilizing PCL membranes also did not show significant differences in membrane surface characteristics, maintaining its characteristics even after exposure to ozone gas [20]. Several studies report contact angles of electrospun PCL with randomly deposited fibers ranging from 122° to 145°. One of the causes of this variation in the contact angle of PCL is the fiber diameters. Additionally, it is observed that there is a significant variation in the molecular weight of the polymers, which can indirectly influence these results [7, 8].

### 3.3. Analysis of Release Profiles—UV–Vis

The UV–Vis absorbance spectra of both nonsterilized and sterilized PCL membranes, Figure 2(a) and 2(b)) respectively, exhibit a clear time‐dependent increase in absorbance, indicating progressive release of UV‐active species into Milli‐Q water. This trend is more pronounced in the nonsterilized samples, where higher absorbance values at each time point suggest a greater concentration of extractable compounds.

Figure 2UV–Vis spectra of PCL membranes: (a) nonsterilized and (b) sterilized with ozone; the inset the linear regression plot of absorbance (max 195 nm) versus time (30–180 min).

**Figure:**
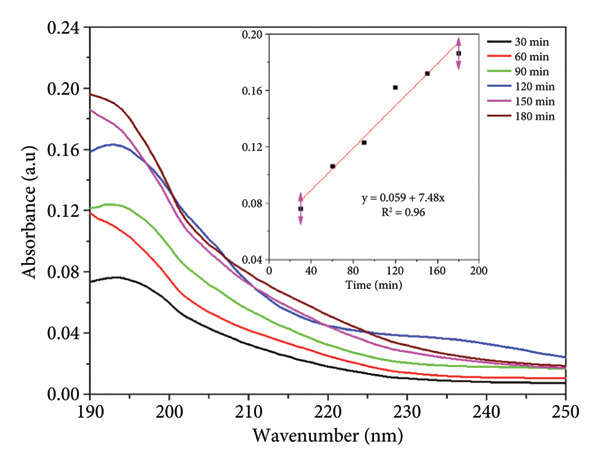
(a)

**Figure:**
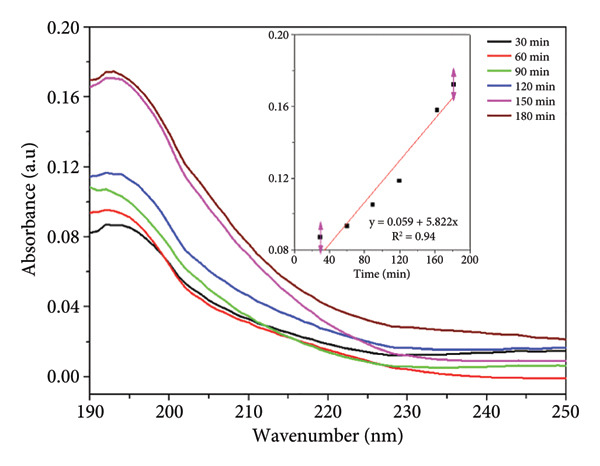
(b)

The progressive increase in absorbance is attributed to the leaching of low‐molecular‐weight species from the PCL matrix, such as residual solvents, unreacted *ε*‐caprolactone monomers, short oligomers, and early degradation products, which are typically observed in semicrystalline aliphatic polyesters. The increase in absorbance is not related to the PCL backbone, which lacks strongly UV‐active chromophores, but rather to the release of low‐molecular‐weight species such as residual solvents, oligomers, or early degradation products that absorb in the deep UV region [14, 21]. This increase is not associated with the PCL backbone, which lacks UV‐active chromophores, but rather with the diffusion of these minor species, remnants of polymer synthesis or processing, into the surrounding aqueous medium. The inset plots in both datasets confirm this behavior, showing a steady, time‐dependent rise in absorbance consistent with a zero‐order leaching profile.

The attenuated response is observed in the sterilized membrane. Figure 2(b) indicates that sterilization may contribute to the removal or stabilization of labile components, thereby enhancing the chemical purity and overall biostability of the material. Such behavior is particularly relevant for biomedical applications, where stability is crucial for ensuring biocompatibility and regulatory compliance [22].

The results indicate that ozone sterilization does not compromise the structural or functional properties of PCL membranes. Thus, these membranes remain applicable in controlled release systems for biomedical applications [23, 24]. The combination of its biocompatibility and tailored degradation profile makes PCL a suitable candidate for drug delivery systems, where precise and predictable release patterns are crucial for therapeutic efficacy [25].

#### 3.3.1. Kinetic Study

The kinetic analysis of UV–Vis absorbance data from PCL membranes immersed in Milli‐Q water revealed that the release of UV‐active compounds over time is best described by a zero‐order kinetic model. For both nonsterilized and sterilized membranes, the zero‐order model yielded higher correlation coefficients (R^2^ = 0.969 and 0.930, respectively) and lower residual errors compared to the first‐order model, particularly in the sterilized samples, where the first‐order fit was notably weaker (R^2^ = 0.704, Table 2).

**Table 2:** Model fitting metrics.

	Model	R^2^	RSS	MSE
Nonsterilized PCL	Zero‐order	0.969	0.000279	4.65 × 10^−5^
	First‐order	0.957	0.000393	6.56 × 10^−5^
Sterilized PCL	Zero‐order	0.930	0.000430	7.16 × 10^−5^
	First‐order	0.704	0.001824	3.04 × 10^−4^

These results suggest that the release mechanism is independent of the concentration of released species and is likely governed by constant rate processes such as surface‐limited diffusion or slow matrix relaxation. This behavior aligns with previously reported zero‐order release kinetics, particularly in cases involving the diffusion‐controlled leaching of low‐molecular‐weight compounds or additives from semicrystalline matrices such as PCL [26].

The lower rate constant observed for sterilized PCL further supports the notion that sterilization reduces the quantity or availability of extractable compounds, thereby enhancing the material’s chemical stability, an essential consideration for biomedical applications where released control and material biocompatibility are critical [14, 21].

### 3.4. Thermal Characterization

The thermogravimetry (TGA) and DSC curves of both before and after sterilization are presented in Figure 3. The TGA curves (Figure 3(a)) reveal that the thermal decomposition of the samples is comparable, occurring in a single stage over 230°C–400°C, with a weight loss exceeding 92%.

Figure 3(a) TGA and (b) DSC spectra of sterilized and nonsterilized PCL membranes.

**Figure:**
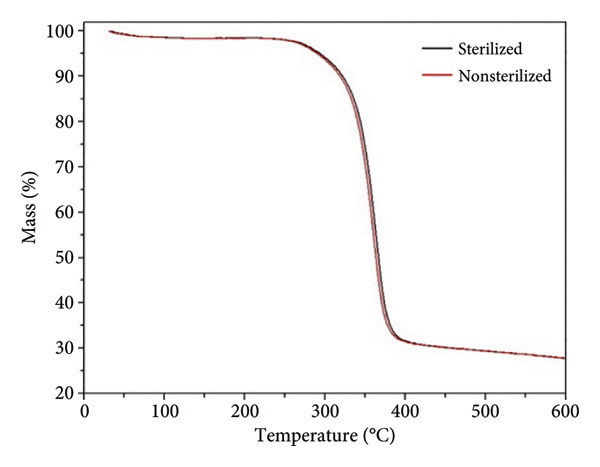
(a)

**Figure:**
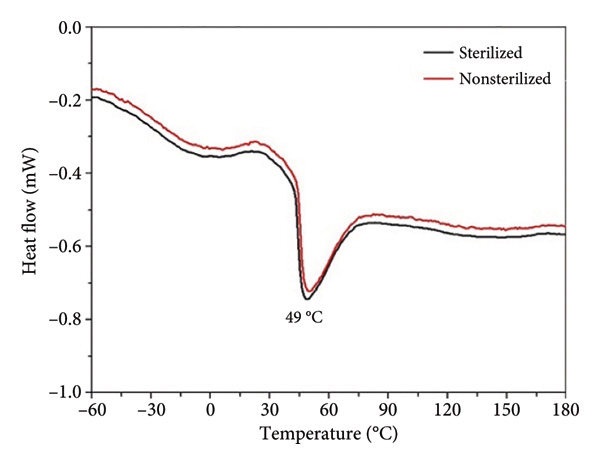
(b)

The DSC thermograms (Figure 3(b)) exhibit a single endothermic peak centered at approximately 49°C, corresponding to the melting temperature (Tm) of PCL. The similarity in the thermal behavior and peak position indicates that ozone sterilization does not significantly alter the thermal stability or chain mobility of the membranes.

The degree of crystallinity (X_c_) of the PCL membranes was calculated from the DSC data using the enthalpy of fusion (ΔH_m_) and Equation (3):

(3) Xc %=ΔHmΔHm°×100,

where ΔH°_m_ = 139.5 J.g^−1^ corresponds to the enthalpy of fusion for 100% crystalline PCL [27]. The calculated crystallinity values were 56.8% for the nonsterilized membrane and 55.9% for the sterilized membrane, showing no statistically significant variation. These results confirm that ozone sterilization preserves the crystalline organization of the polymer chains. The maintenance of crystallinity after sterilization agrees with the similar melting temperatures observed for both samples and with previous reports indicating that ozone treatment does not induce molecular rearrangements or chain scission in electrospun PCL fibers [7, 19, 28].

### 3.5. Spectroscopy Characterization

Figure 4 presents the FTIR measurement of sterilized and nonsterilized PCL membranes, showing the most relevant peaks and their respective assignments. The obtained spectra were similar, and no additional peaks were observed after sterilization, indicating the presence of the characteristic functional groups of PCL. A band is observed at 2869 cm^−1^, corresponding to C–H stretching. The band at 1722 cm^−1^ is characteristic of the stretching of the C=O and ester functional groups (C‐O bond stretching) at 1242 cm^−1^, as reported by Khosravi [20].

Figure 4(a) FTIR, (b) Raman spectra, and (c) stress–strain curves of PCL membrane sterilized and nonsterilized.

**Figure:**
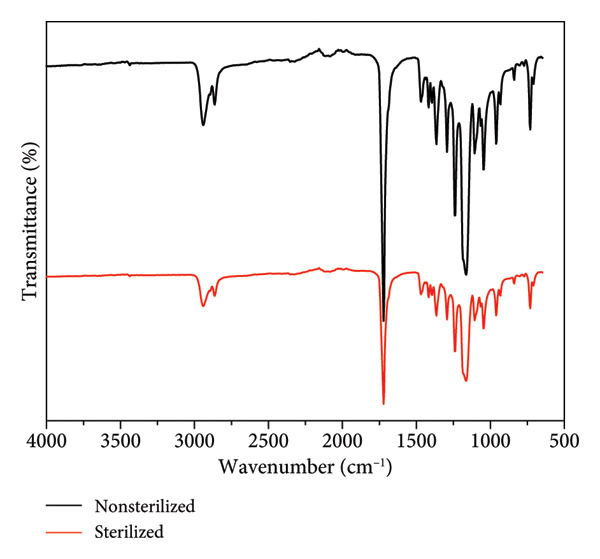
(a)

**Figure:**
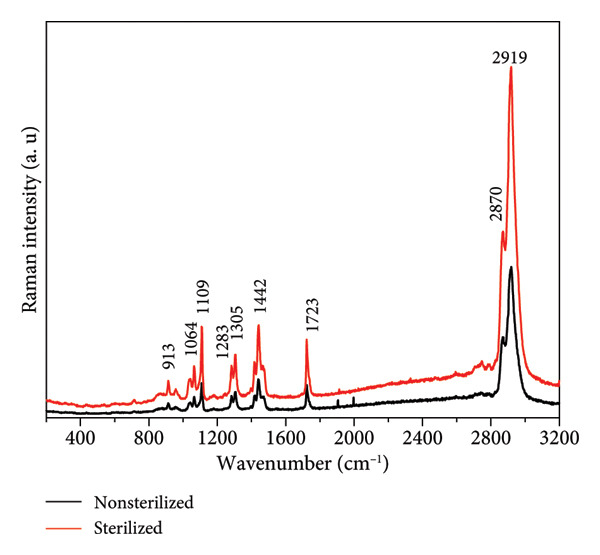
(b)

**Figure:**
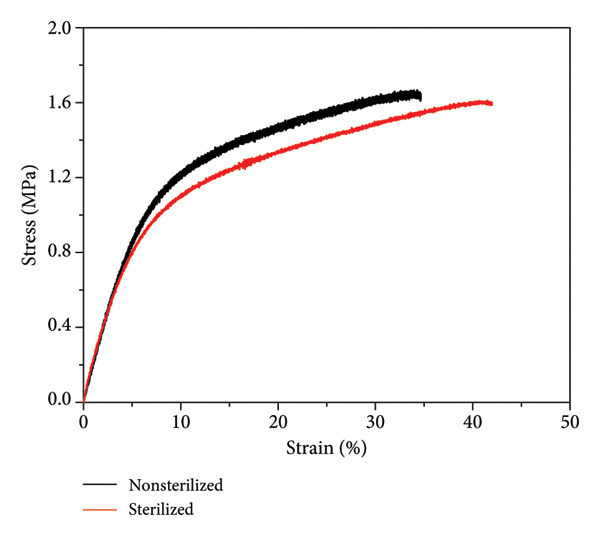
(c)

The results indicate the absence of new organic functions, suggesting that the chemical composition of the membranes remained unchanged. Studies comparing various sterilization techniques across different polymers evaluated the characteristics of electrospun polymers before and after sterilization using FTIR and showed that ozone sterilization preserved the polymer’s chemical characteristics [29].

Figure 4(b) shows the comparative Raman spectra of PCL sterilized and nonsterilized. Considering the intense overlap of the broad bands arising from the asymmetric and symmetric stretching vibrations of CH_2_ (appearing at 2918 and 2870 cm^−1^, respectively). No differences in band positions were observed between the spectra obtained on the sterilized and nonsterilized membranes. The positions of the bands and their assignments are presented in Table 2. The bands indicating PCL appear at 1722 cm^−1^ (C=O), 1284 cm^−1^ (ωCH_2_), and 1110 cm^−1^ (C–O–C) [16, 30] (Table 3).

**Table 3:** Raman bands of PCL membranes.

Raman bands in this study (cm^−1^)	Reported in references [16, 30]	Assignment
914	912	*ν*(C–COO)
955	957	*ν*(C–COO)
1039	1036	*ν*(COC)
1063	1065	*ν*(COC)
1110	1108	*ν*(COC)
1284	1284	*ω*(CH_2_)
1304	1304	*ω*(CH_2_)
1420	1417	*δ*(CH_2_)
1440	1440	*δ*(CH_2_)
1471	1465	*δ*(CH_2_)
1722	1723	*ν*(C = O)
2870	2869	*ν*(CH_2_)
2918	2919	*ν*(CH_2_)

On the other hand, the bands at 1733 cm^−1^ (coming from the C=O stretching) and 1097 cm^−1^ (C–O–C), characteristic of the amorphous domains of PCL, appear as a broadening of the bands that characterize the crystallinity of the material and are distinguished by applying the spectral curve fitting method. The group of peaks with the band at approximately 1440 cm^−1^ (δCH_2_) and the peak at 914 cm^−1^ is also derived from the crystalline domain. The band at 1304 cm^−1^ (coupled with the band at 1284 cm^−1^) results from the bending vibration of the CH_2_ groups (ωCH_2_) and is present in the amorphous and crystalline phases of the polymer [16]. The characteristic bands of crystalline domains are relatively narrow and intense.

No spectral changes were observed that would indicate the emergence of new organic functional groups, confirming that the chemical composition of the membranes remained unaltered. Comparative studies on different sterilization techniques across various polymers, which assessed the properties of electrospun polymers before and after sterilization using FTIR and Raman spectroscopy, demonstrated that ozone sterilization preserved the chemical characteristics of the polymers [28, 29].

Figure 4(c) presents the stress–strain curves for nonsterilized and sterilized PCL membranes. Both electrospun membrane samples exhibited similar mechanical behavior, characterized initially by elastic deformation followed by plastic deformation until failure. The comparable profiles indicate that the ozone sterilization process does not significantly affect the mechanical integrity of the membranes. The maximum stress values were 1.67 MPa for the nonsterilized sample and 1.61 MPa for the sterilized sample. Similarly, Young’s modulus values were 23.15 MPa (nonsterilized) and 25.68 MPa (sterilized). These results, consistent with Rediguieri et al. (2017), confirm that ozone treatment effectively preserves the mechanical properties of electrospun PCL membranes [16].

### 3.6. Sterilization Study

**Figure 5:**
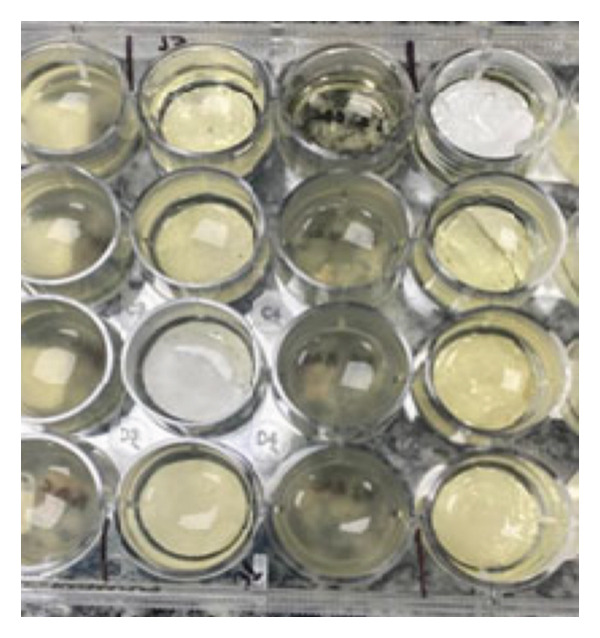
Culture media for the sterility study of electrospun membranes.

The sterility tests indicated no turbidity in the growth media and no formation of bacterial or fungal colonies on the culture plates, as shown in Figure 5. These results confirm the absence of microbial contamination after the ozone sterilization process. The nonsterilized membranes were evaluated, and no bacterial or fungal growth was observed.

The absence of microbial contamination is crucial for conducting biological assays, as the presence of bacteria or fungi would compromise experiment viability, rendering the results unreliable. Furthermore, contamination could compromise membrane integrity, alter experimental parameters, and hinder data interpretation. Therefore, it can be concluded that the ozone sterilization process was effective for the electrospun polymer membranes evaluated in this study, ensuring the sterile conditions necessary for reliable biomedical experiments.

Rediguieri et al. [16, 23] investigated the impact of ozone sterilization on poly(lactic‐co‐glycolic acid) (PLGA) and PCL nanofiber scaffolds. After incubation in tryptic soy broth (TSB) and thioglycollate broth, the media remained clear after 14 days, indicating no microbial growth and confirming the scaffolds′ sterility, achieved with just two ozone pulses.

### 3.7. Evaluation of the Viability and Cytotoxicity of Human Lung Fibroblasts (MRC‐5)

**Figure 6:**
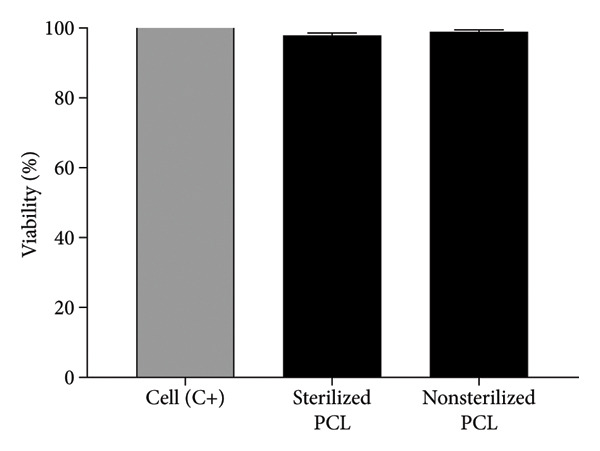
Percentage of cell viability against human fibroblast cells (MRC5).

Cell viability and cytotoxicity of the membranes were evaluated based on the previously characterized release profiles for each membrane. This procedure is crucial to determine the biological safety and compatibility of the membranes with biological systems. Furthermore, these analyses provide indispensable information on the impact of the sterilization process on cellular responses, ensuring that the material does not cause adverse effects and is suitable for biomedical and biotechnological applications. In Figure 6, cell viability percentages are presented, compared to the control (100% viable or living cells). The results demonstrate that nonsterilized PCL exhibited a cell viability of 98.95%, while sterilized PCL showed a viability of 97.83% concerning MRC‐5 cells. The absence of significant differences between sterilized and nonsterilized membranes confirms that ozone treatment preserved the intrinsic biocompatibility of PCL, ensuring sterility without cytotoxicity.

Rediguieri et al. [16] evaluated the impact of ozone sterilization on the properties and cell compatibility of electrospun PCL membranes using the L929 fibroblast cell line. The study demonstrated that ozone gas sterilization effectively sterilized the membranes while preserving most of their initial characteristics. The fibers remained effective in the treatment, enhancing cell proliferation and maintaining cell viability. While recent studies have reported the antiproliferative and apoptotic effects of ozone when combined with compounds like doxorubicin [25], the findings of this study suggest that ozone treatment remains a viable technique for sterilizing electrospun polymers. This is particularly relevant as other sterilization methods can significantly damage these membranes.

### 3.8. Morphological Evaluation and Cell Adhesion Studies

**Figure 7:**
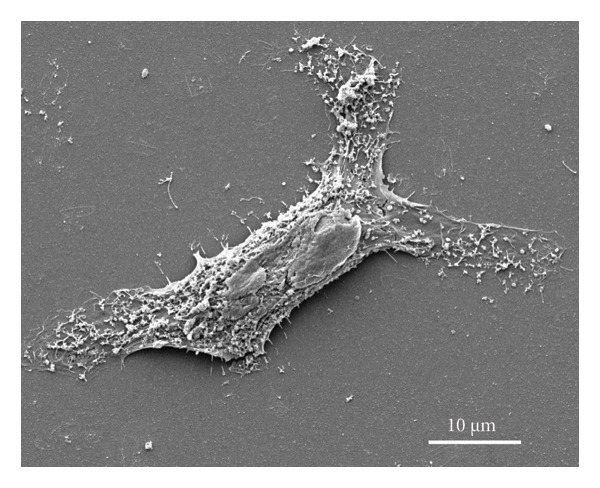
SEM micrograph of the control sample.

Figure 7 illustrates the SEM images of a single cell adhered to the surface, exhibiting a flattened morphology with extended pseudopodia that facilitate the adhesion and anchorage of fibroblasts (MRC‐5) [9]. The micrograph reveals detailed surface interactions between the cell and the underlying substrate, characterized by the formation of filopodia and lamellipodia, indicative of effective cellular adhesion. However, it is essential to note that the SEM sample preparation process induced cellular stress, which may have partially compromised the integrity of the observed cells.

The micrographs in Figure 8 illustrate cell adhesion on samples with (Figure 8(a)) nonsterilized PCL nanofibers and (Figure 8(b)) sterilized PCL nanofibers after 48 h of incubation. The images reveal that PCL nanofibers provide a suitable surface for cell adhesion and anchorage. No significant differences in cell anchorage are observed between the sterilized and nonsterilized membranes, as both show well‐anchored and numerous cells.

Figure 8Cell adhesion of the MRC‐5 cell line on samples of (a) nonsterilized PCL nanofibers and (b) sterilized PCL nanofibers.

**Figure:**
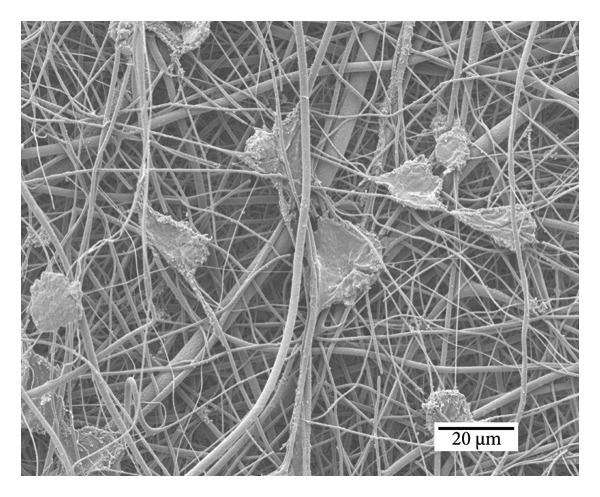
(a)

**Figure:**
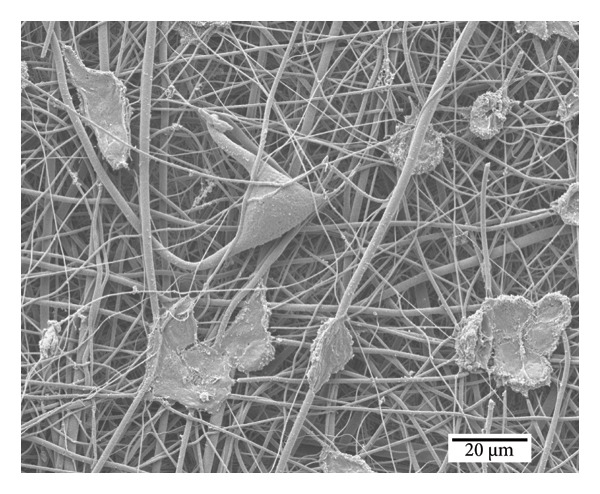
(b)

Azizoglu et al. [31] studied cell proliferation on PCL membranes for wound healing applications using the MRC‐5 cell line, reporting a cell viability of approximately 144%. Similarly, a viability rate of 145% was observed with the immortalized human keratinocyte HACAT cell line. However, their study did not specify the sterilization method used on the fibers. In contrast, our work emphasizes ozone as an effective and reliable sterilization technique for materials intended for biomedical applications, addressing this critical aspect to ensure the membranes′ suitability and safety for clinical use.

Table 4 summarizes the effects of different sterilization methods on electrospun PCL scaffolds, highlighting the main structural and physicochemical changes reported in the literature. The comparison between treated and untreated samples provides a useful reference for evaluating how each method influences fiber morphology, surface properties, and mechanical performance. This contextual analysis underscores the relevance of exploring ozone sterilization as an alternative technique capable of maintaining the functional integrity of PCL scaffolds while ensuring effective microbial inactivation.

**Table 4:** Effects of sterilization on PCL electrospun scaffolds observed after comparing treated samples to nontreated ones.

Sterilization method	Preserved properties	Altered properties^a^	References
Autoclave	N/A	Melted fibers	[32]
Gamma radiation	Morphology, FTIR spectra	↓ tensile strength, ↓ molecular weight, ↑ melting temperature	[33]
Gamma radiation	N/A	↓ tensile strength, ↑ crystallinity %, FTIR spectra, ↓water contact angle, ↑ cell coverage after 24 h of seeding	[34]
Ethylene oxide	N/A	Translucent and brittle, ↓ water contact angle, impermeable to water	[32]
Peracetic acid (PAA)	Mechanical properties	PAA diluted in DI: fiber fusion, fiber breakage, and ↑ fiber diameter PAA diluted in EtOH: ↓ fiber diameter ↓ water contact angle, ↓ permeability	[32]
Plasma	Morphology	↓ water contact angle	[35]

^a^↑‐ Increase, ↓ ‐Decrease.

## 4. Conclusion

This study provides comprehensive insights into the development and characterization of electrospun PCL membranes, demonstrating their efficacy and versatility for biomedical applications. The membranes exhibited uniformly distributed fibers with diameters ranging from 0.3 to 0.5 μm. Chemical analyses, including FTIR and Raman spectroscopies, confirmed the presence of characteristic functional groups of PCL and revealed no significant chemical alterations resulting from ozone sterilization. This underscores the method’s capability to effectively sterilize membranes without impacting their structural or chemical integrity.

The release behavior of UV‐active compounds from PCL membranes followed a zero‐order release profile characterized by a linear increase in absorbance over time, indicating a constant release rate independent of the concentration of extractable species in the medium. This suggests that surface‐limited or diffusion‐controlled mechanisms dominate the leaching process. The zero‐order model provided an excellent fit to the experimental data, particularly for the nonsterilized membranes (R^2^ = 0.969). Sterilized membranes also followed the same kinetic trend, albeit with a lower release rate. These findings highlight the stability enhancement imparted by sterilization and reinforce the suitability of PCL membranes for biomedical applications.

Furthermore, thermal characterizations through DSC and TGA demonstrated the stability of the PCL membranes, with no noticeable differences between sterilized and nonsterilized samples. The hydrophobic nature of the material was maintained, with contact angles remaining consistent at approximately 123°. Biocompatibility tests revealed high cell viability (> 97%) and excellent support for cell adhesion and proliferation, highlighting the material’s suitability for tissue engineering and other medical applications.

The study also demonstrated that PCL membranes sterilized with ozone retain their morphological, chemical, and functional properties, making them suitable candidates for various applications, including wound dressings and drug delivery systems. Future advancements may involve incorporating bioactive substances or composite materials to enhance their functionality further. For instance, blending PCL with polymers such as cellulose acetate, chitosan, or hyaluronic acid could significantly improve mechanical and thermal properties. Additionally, integrating metallic nanoparticles, such as gold and silver, could provide antimicrobial and anti‐inflammatory benefits, offering significant advantages in wound healing and infection prevention.

Overall, the findings of this study pave the way for the broader application of electrospun PCL membranes in biomedical engineering, emphasizing their potential as scalable, biocompatible, and multifunctional scaffolds for advanced therapeutic solutions.

## Conflicts of Interest

The authors declare no conflicts of interest.

## Funding

This work was supported by the Fundação de Amparo à Pesquisa do Estado do Amazonas (FAPEAM), the Coordenação de Aperfeiçoamento de Pessoal de Nível Superior (CAPES), the Universidade Federal do Amazonas (UFAM), and the Conselho Nacional de Desenvolvimento Científico e Tecnológico (CNPq).

## Data Availability

The data that support the findings of this study are available from the corresponding author upon reasonable request.

## References

[1] Farias J. , Ricardo J. , Cunha J. et al., Electrospun Poly(ɛ-Caprolactone) Membranes Modified with Heparin and Essential Fatty Acids for Biomedical Applications, Journal of Applied Polymer Science. (2024) 1, no. 34, 10.1002/app.55853.

[2] Sanfelice R. C. , Pavinatto A. , and Corrêa D. S. , Introdução à Nanotecnologia, Nanotecnologia Aplicada a Polímeros, 2022, 10.5151/9786555502527-01.

[3] Santos C. G. , Roggia I. , Fernandes L. da S. , and Raffin R. P. , Uso de Blendas Poliméricas Em Nano e Microencapsulação, Disciplinarum Scientia. Série: Naturais e Tecnológicas, Santa Maria. (2015) 16, no. 2.

[4] Araújo B. A. , Silva De Freitas L. , Kelly K. et al., The Application of Biodegradable Polymers as a Sustainable Alternative, Research, Society and Development. (2021) 10, no. 9.

[5] Rezvani Ghomi E. , Khalili S. , Nouri Khorasani S. , Esmaeely Neisiany R. , and Ramakrishna S. , Wound Dressings: Current Advances and Future Directions, Journal of Applied Polymer Science. (2019) 136, no. 27, 10.1002/app.47738, 2-s2.0-85063983021.

[6] Gu L. , Yu C. , Chen K. et al., Simultaneous Degradation of Pharmaceutical Contaminants and Sterilization in Real Hospital Wastewater by a Highly Efficient Electrocatalytic Ozonation Process: Performance, Mechanism and Application, Chemical Engineering Journal. (2024) 491, 10.1016/j.cej.2024.152020.

[7] Galante R. , Ghisleni D. , Paradiso P. et al., Sterilization of Silicone-Based Hydrogels for Biomedical Application Using Ozone Gas: Comparison with Conventional Techniques, Materials Science and Engineering: C. (2017) 78, 389–397, 10.1016/j.msec.2017.04.073, 2-s2.0-85018518366. 28575999

[8] Moraes Segundo J. D. P. , Moraes M. O. S. , Brito W. R. et al., Synthesis of Gallic Acid Imprinted Polymer and Incorporation in Poly(Caprolactone) Mat via Electrospinning, Express Polymer Letters. (2021) 15, no. 7, 654–665, 10.3144/EXPRESSPOLYMLETT.2021.55.

[9] Ding S. M. , Lu J. F. , Edoo M. I. A. et al., MRC-5 Cancer-Associated Fibroblasts Influence Production of Cancer Stem Cell Markers and Inflammation-Associated Cell Surface Molecules, in Liver Cancer Cell Lines, International Journal of Medical Sciences. (2019) 16, no. 8, 1157–1170, 10.7150/ijms.34758, 2-s2.0-85072170421. 31523179 PMC6743285

[10] Moraes Segundo J. de D. P. de , Oneide Silva de Moraes M. , Brito W. R. , and d’Ávila M. A. , Incorporation of Molecularly Imprinted Polymer Nanoparticles in Electrospun Polycaprolactone Fibers, Materials Letters. (2020) 275, 10.1016/j.matlet.2020.128088.

[11] Hotaling N. A. , Bharti K. , Kriel H. , and Simon C. G. , DiameterJ: a Validated Open Source Nanofiber Diameter Measurement Tool, Biomaterials. (2015) 61, 327–338, 10.1016/j.biomaterials.2015.05.015, 2-s2.0-84939167165. 26043061 PMC4492344

[12] Higuchi T. , Mechanism of Sustained‐Action Medication. Theoretical Analysis of Rate of Release of Solid Drugs Dispersed in Solid Matrices, J Pharm Sci. (1963) 52, no. 12, 1145–1149, 10.1002/jps.2600521210, 2-s2.0-0344893332. 14088963

[13] Siepmann J. , Modeling of Drug Release from Delivery Systems Based on Hydroxypropyl Methylcellulose (HPMC), Advanced Drug Delivery Reviews. (2001) 48, no. 2–3, 139–157, 10.1016/S0169-409X(01)00112-0, 2-s2.0-0035844738. 11369079

[14] Woodruff M. A. and Hutmacher D. W. , The Return of a Forgotten Polymer—Polycaprolactone in the 21st Century, Progress in Polymer Science. (2010) 35, no. 10, 1217–1256, 10.1016/j.progpolymsci.2010.04.002, 2-s2.0-77957588918.

[15] Mohamed R. M. and Yusoh K. , A Review on the Recent Research of Polycaprolactone (PCL), Advanced Materials Research. (2015) 1134, 249–255, 10.4028/www.scientific.net/AMR.1134.249.

[16] Rediguieri C. F. , De Bank P. A. , Zanin M. H. A. et al., The Effect of Ozone Gas Sterilization on the Properties and Cell Compatibility of Electrospun Polycaprolactone Scaffolds, Journal of Biomaterials Science, Polymer Edition. (2017) 28, no. 16, 1918–1934, 10.1080/09205063.2017.1358549, 2-s2.0-85026513400. 28737465

[17] De Souza N. L. G. D. , Cavallini G. S. , Alves T. T. , Pereira M. M. , Mello Brandão H. de , and Oliveira L. F. C. de. , Compatibility and Cytotoxicity of Poly(ε-Caprolactone)/Polypyrrole-Block-Poly(ε-Caprolactone) Blend Films in Fibroblast Bovine Cells, Polimeros. (2024) 34, no. 1, 10.1590/0104-1428.20230082.

[18] Antunes Gonçalves N. , Nanofibras de Poli(ɛ-Caprolactona) e Poli(Óxido de Etileno): Fabricação Pela Técnica de Eletrofiação e Efeitos Radiolíticos, Programa de Pós-Graduação em Tecnologias Energéticas e Nucleares. (2015) Universidade Federal de Pernambuco, Recife–Pernambuco–Brasil.

[19] Dabasinskaite L. , Krugly E. , Baniukaitiene O. et al., The Effect of Ozone Treatment on the Physicochemical Properties and Biocompatibility of Electrospun Poly(ε)Caprolactone Scaffolds, Pharmaceutics. (2021) 13, no. 8, 10.3390/pharmaceutics13081288. PMC8400338 34452249

[20] Wang Y. , Rodriguez-Perez M. A. , Reis R. L. , and Mano J. F. , Thermal and Thermomechanical Behaviour of Polycaprolactone and Starch/Polycaprolactone Blends for Biomedical Applications, Macromolecular Materials and Engineering. (2005) 290, no. 8, 792–801, 10.1002/mame.200500003, 2-s2.0-24644465661.

[21] Dash T. K. and Konkimalla V. B. , Poly-є-Caprolactone Based Formulations for Drug Delivery and Tissue Engineering: A Review, Journal of Controlled Release. (2012) 158, no. 1, 15–33, 10.1016/j.jconrel.2011.09.064, 2-s2.0-84861651995. 21963774

[22] Anderson J. M. and Shive M. S. , Biodegradation and Biocompatibility of PLA and PLGA Microspheres, Advanced Drug Delivery Reviews. (1997) 28, no. 1, 5–24, 10.1016/S0169-409X(97)00048-3, 2-s2.0-0343247711. 10837562

[23] Rediguieri C. F. , Sassonia R. C. , Dua K. , Kikuchi I. S. , and de Jesus Andreoli Pinto T. , Impact of Sterilization Methods on Electrospun Scaffolds for Tissue Engineering, European Polymer Journal. (2016) 82, 181–195, 10.1016/j.eurpolymj.2016.07.016, 2-s2.0-84979916687.

[24] Horakova J. , Klicova M. , Erben J. et al., Impact of Various Sterilization and Disinfection Techniques on Electrospun Poly-ϵ-Caprolactone, ACS Omega. (2020) 5, no. 15, 8885–8892, 10.1021/acsomega.0c00503. 32337451 PMC7178787

[25] Bhadran A. , Shah T. , Babanyinah G. K. et al., Recent Advances in Polycaprolactones for Anticancer Drug Delivery, Pharmaceutics. (2023) 15, no. 7, 10.3390/pharmaceutics15071977. PMC10385458 37514163

[26] Langer R. , New Methods of Drug Delivery, Science. (1990) 249, no. 4976, 1527–1533, 10.1126/science.2218494, 2-s2.0-0025047522. 2218494

[27] Fernández-Tena A. , Pérez-Camargo R. A. , Coulembier O. et al., Effect of Molecular Weight on the Crystallization and Melt Memory of Poly(ε-Caprolactone) (PCL), Macromolecules. (2023) 56, no. 12, 4602–4620, 10.1021/acs.macromol.3c00234.

[28] Khosravi A. , Ghasemi-Mobarakeh L. , Mollahosseini H. et al., Immobilization of Silk Fibroin on the Surface of PCL Nanofibrous Scaffolds for Tissue Engineering Applications, Journal of Applied Polymer Science. (2018) 135, no. 37, 10.1002/app.46684, 2-s2.0-85050387037.

[29] Talita Almeida Vida DE Brito , Preparação e Caracterização de Nanofibras Da Blenda PLLA/PCL Obtidas Pelos Processos de Eletrofiação e Rotofiação, 2013, Campinas-Brasil.

[30] Rodrigues A. S. , Bioresorbable Polymers for Tissue Engineering, Tissue Engineering. (2011) 18, no. 18.

[31] Azizoğlu G. A. , Azizoğlu E. , Barker T. H. , and Özer Ö. , Single and Multi-Dose Drug Loaded Electrospun Fiber Mats for Wound Healing Applications, Journal of Drug Delivery Science and Technology. (2023) 81, 10.1016/j.jddst.2023.104168.

[32] Yoganarasimha S. , Trahan W. R. , Best A. M. et al., Peracetic Acid: a Practical Agent for Sterilizing Heat-Labile Polymeric Tissue-Engineering Scaffolds, Tissue Engineering Part C Methods. (2014) 20, no. 9, 714–723, 10.1089/ten.tec.2013.0624, 2-s2.0-84906960706. 24341350 PMC4152794

[33] Bosworth L. A. , Gibb A. , and Downes S. , Gamma Irradiation of Electrospun Poly(Ε‐caprolactone) Fibers Affects Material Properties but Not Cell Response, Journal of Polymer Science Part B: Polymer Physics. (2012) 50, no. 12, 870–876, 10.1002/polb.23072, 2-s2.0-84860802819.

[34] Augustine R. , Saha A. , Jayachandran V. P. , Thomas S. , and Kalarikkal N. , Dose-Dependent Effects of Gamma Irradiation on the Materials Properties and Cell Proliferation of Electrospun Polycaprolactone Tissue Engineering Scaffolds, International Journal of Polymeric Materials and Polymeric Biomaterials. (2015) 64, no. 10, 526–533, 10.1080/00914037.2014.977900, 2-s2.0-84922749765.

[35] Dai Y. , Xia Y. , Chen H.-B. et al., Optimization of Sterilization Methods for Electrospun Poly(ε-Caprolactone) to Enhance Pre-Osteoblast Cell Behaviors for Guided Bone Regeneration, Journal of Bioactive and Compatible Polymers. (2016) 31, no. 2, 152–166, 10.1177/0883911515598795, 2-s2.0-84958523861.

